# The Curious Case of the Cephalopod Parasites

**DOI:** 10.3201/eid2608.AC2608

**Published:** 2020-08

**Authors:** Byron Breedlove

**Affiliations:** Centers for Disease Control and Prevention, Atlanta, Georgia, USA

**Keywords:** art science connection, emerging infectious diseases, art and medicine, about the cover, public health, malaria, the curious case of the cephalopod parasites, parasites, parasitic diseases, A. E. d’Audebard de baron de Férussac, Alcide Dessalines d’Orbigny, natural, general and particular history of the acetabuiferous cephalopods living, cephalopods, octopus, neglected tropical diseases

**Figure:**
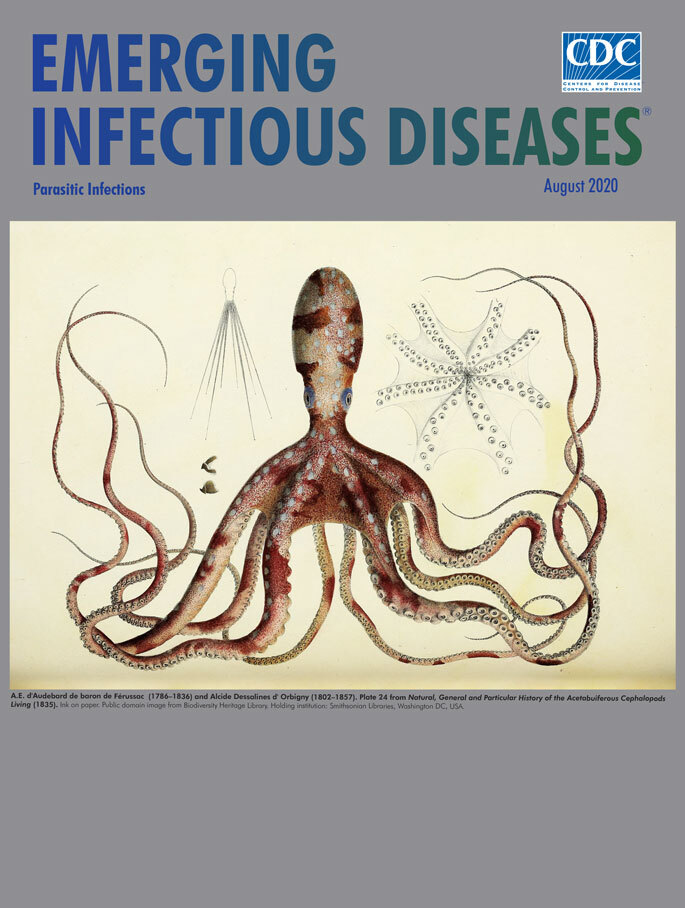
**A. E. d’Audebard de baron de Férussac (1786–1836) and Alcide Dessalines d’Orbigny (1802–1857). Plate 24 from *Natural, general and particular history of the acetabuiferous cephalopods living* (1835).** Ink on paper. Public domain image from Biodiversity Heritage Library. Holding institution: Smithsonian Libraries, Washington, DC, USA.

This month’s cover illustration of an octopus comes from the book *Natural, General and Particular History of the Acetabuiferous Cephalopods Living* (see the bibliography for the full title). This voluminous study of cephalopods was completed by a pair of 19th century French naturalists, though not exactly as collaborators. A. 

E. d’Audebard de baron de Férussac wrote the introduction and the first 11 parts; Alcide Dessalines d’Orbigny revised and completed the book. Férussac, a professor of geography and statistics at the École d'état-major in Paris, is now chiefly recognized for his studies of molluscs. D’Orbigny, a professor of paleontology at the Paris Muséum National d’Histoire Naturelle, collected natural specimens from South America and corresponded with Charles Darwin. 

Completed in the 1830s, this detailed illustration does not identify the species of octopus represented, though it is most likely a common octopus, which would have been 1–3 feet long and weighed 10–20 pounds. Nonetheless, it displays the animal’s characteristic features: bulbous head, wide-angled eyes that provide a panoramic view, 8 whip-like arms festooned with suckers, and mottled and flecked colors. One companion image resembling a multipronged compass protractor reveals the scale and reach of the straightened arms, and on first glance, a second drawing of the creature’s underside could pass for a splayed umbrella. 

An undeserved reputation for ferocity and belligerence has been foisted on the octopus. In the first century ce, Pliny the Elder decried, “no animal is more savage in causing the death of a man in the water.” The air of mystery attached to these intelligent, curious creatures befits them, no doubt spurred by their physical appearance. Anthropologist Roland Burrage Dixon references a Hawaiian creation myth that describes a primordial ocean in which “swims the octopus, the lone survivor from an earlier world.” 

Given their outward appearance, labeling these creatures as alien or otherworldly does not seem farfetched. Examining their anatomy does not dispel those notions. Their arms, which contain more neurons than their brains, collect and convey an array of sensory information and may even have distinct personalities. Of their three hearts, two move blood from their gills and the third circulates their blood. Their blue blood is tinted by the copper-transporting protein hemocyanin, which is more efficient than hemoglobin for transporting oxygen in frigid and oxygen-poor ocean water. 

Octopuses are acknowledged for their adeptness at solving intricate problems and using tools, curiosity, and even mischief. Able to mimic colors and textures and to squeeze into astonishingly compact spaces, these cephalopods excel as both hunters and survivalists. Science writer Katherine Harmon Courage writes, “The boneless octopus must avoid becoming lunch for sharks, eels, fish and even killer whales. But not all of the organisms that feed on octopuses are such charismatic megafauna.” 

Parasitic organisms occur in all animal species, are as diverse as their host species, and derive their sustenance, during at least part of their lifecycles, at the expense of their hosts. This ubiquitous template of coexistence has persisted and evolved and given rise to euryxenous parasites that infect a spectrum of unrelated hosts; stenoxenous parasites that prefer closely related hosts; and oioxenous parasites that limit themselves to single species of host. Among the latter two types is a phylum of highly specialized parasites known as Rhombozoa or Dicyemida, which dwell only in the kidneys of cephalopods.

Some of the estimated 250–300 known species of octopus are so similar that researchers differentiate them by examining those parasites. Courage writes, “These microorganisms are often unique not just to the octopus but also to a particular species of octopus. In fact, these very specific, kidney-dwelling species can even be used to tell one octopus species from another. (With such variable physical attributes, octopus specimens can be difficult to parse.)”

Parasites can cause a range of diseases in humans, domestic animals, and wildlife. Myriad parasitic infections range from asymptomatic to mild to severe to fatal. They are encountered by hosts of every species on every continent and in every body of water. A confluence of factors―including deforestation, urbanization, and development, international travel and tourism, contemporary agricultural practices, and displacement of wildlife, sanitation problems, and growing antimicrobial and insecticide resistance―encumbers efforts to control parasitic infections. Discerning the abundance, diversity, specialization, and history of parasitic organisms, such as the curious case of the finicky Rhombozoa, may yield information with potential to improve our understanding of other parasitic infections.
